# A new paradigm of DNA synthesis: three-metal-ion catalysis

**DOI:** 10.1186/s13578-016-0118-2

**Published:** 2016-09-06

**Authors:** Wei Yang, Peter J. Weng, Yang Gao

**Affiliations:** Laboratory of Molecular Biology, NIDDK, National Institutes of Health, 9000 Rockville Pike, Bethesda, MD 20892 USA

## Abstract

Enzyme catalysis has been studied for over a century. How it actually occurs has not been visualized until recently. By combining in crystallo reaction and X-ray diffraction analysis of reaction intermediates, we have obtained unprecedented atomic details of the DNA synthesis process. Contrary to the established theory that enzyme-substrate complexes and transition states have identical atomic composition and catalysis occurs by the two-metal-ion mechanism, we have discovered that an additional divalent cation has to be captured en route to product formation. Unlike the canonical two metal ions, which are coordinated by DNA polymerases, this third metal ion is free of enzyme coordination. Its location between the α- and β-phosphates of dNTP suggests that the third metal ion may drive the phosphoryltransfer from the leaving group opposite to the 3′-OH nucleophile. Experimental data indicate that binding of the third metal ion may be the rate-limiting step in DNA synthesis and the free energy associated with the metal-ion binding can overcome the activation barrier to the DNA synthesis reaction.

**Electronic supplementary material:**

The online version of this article (doi:10.1186/s13578-016-0118-2) contains supplementary material, which is available to authorized users.

## Background

In 1926 James B. Sumner purified and crystallized the first enzyme urease from jack bean and found that the crystalline form of urease catalyzed the breakdown of urea to ammonium and carbon dioxide [1]. For this feat Sumner received the Nobel Prize in Chemistry in 1946. As early as 1835, the concept of catalysis was suggested by Jacob Berzelius, and in 1935 Henry Eyring, Meredith Gwynne Evans and Michael Polanyi proposed the transition state theory (TST), in which reactants and activated transition state complexes co-exist in a quasi-equilibrium. Based on the TST, activation energies for enzyme catalysis are routinely calculated from experimentally observed reaction rates [2]. It has been taken for granted that enzymes catalyze reactions by stabilizing the transition state and thus reducing the activation energy and accelerating the reaction rate.

Owing to the transient nature of the transition state and technical difficulties of obtaining precise temporal and spatial details of a dynamic process, the exact details of any chemical reaction and how enzymes reduce energy barriers without consuming anything remain unknown. The questions regarding where the activation energy for catalysis, reduced yet still necessary, comes from and how enzymes stabilize the transition state have remained unanswered even after decades of experiments and theoretical calculations. Moreover, in practice many attempts to create artificial enzymes based on the assumed catalytic role of stabilizing transition states have met little success [3–5].

Kinetic rate analyses, particularly at pre-steady state, have generated a wealth of information about enzyme catalysis, intermediate steps and rates of their occurrence [6, 7]. However, various tricks have to be applied to alter reaction processes so that individual intermediate steps can be analyzed and their occurrence rate measured [8–13]. Similar to kinetic studies, to obtain three-dimensional structures of enzyme-substrate complexes, chemical reactions have to be manipulated and stopped by using non-reactive substrate mimics, enzymes inactivated by mutations, or non-permissible cofactors [14–19]. Assuming identical chemical compositions throughout the reaction process as suggested by the transition-state theory, transition states and reaction processes are then reconstructed and modeled based on experimentally measured rates and trapped enzyme-substrate or enzyme-product complexes by rearranging atoms, protons and electrons [20–23].

In recent years, we have developed a new approach to study the kinetics of DNA synthesis reactions and directly visualize reaction intermediates by in crystallo reaction and time-resolved X-ray crystallography. Although at a relatively low temporal resolution (tens of seconds), the actual reaction process is captured at atomic resolution [24]. In this review article we present the surprising finding of a third metal ion that is absent in the enzyme-substrate complex and captured by substrates in the transition state [25]. Binding of this additional third metal ion may provide sufficient activation energy to overcome the barrier to product formation.

## Review

### Limitation of existing methods of studying catalysis

**Fig. 1:**
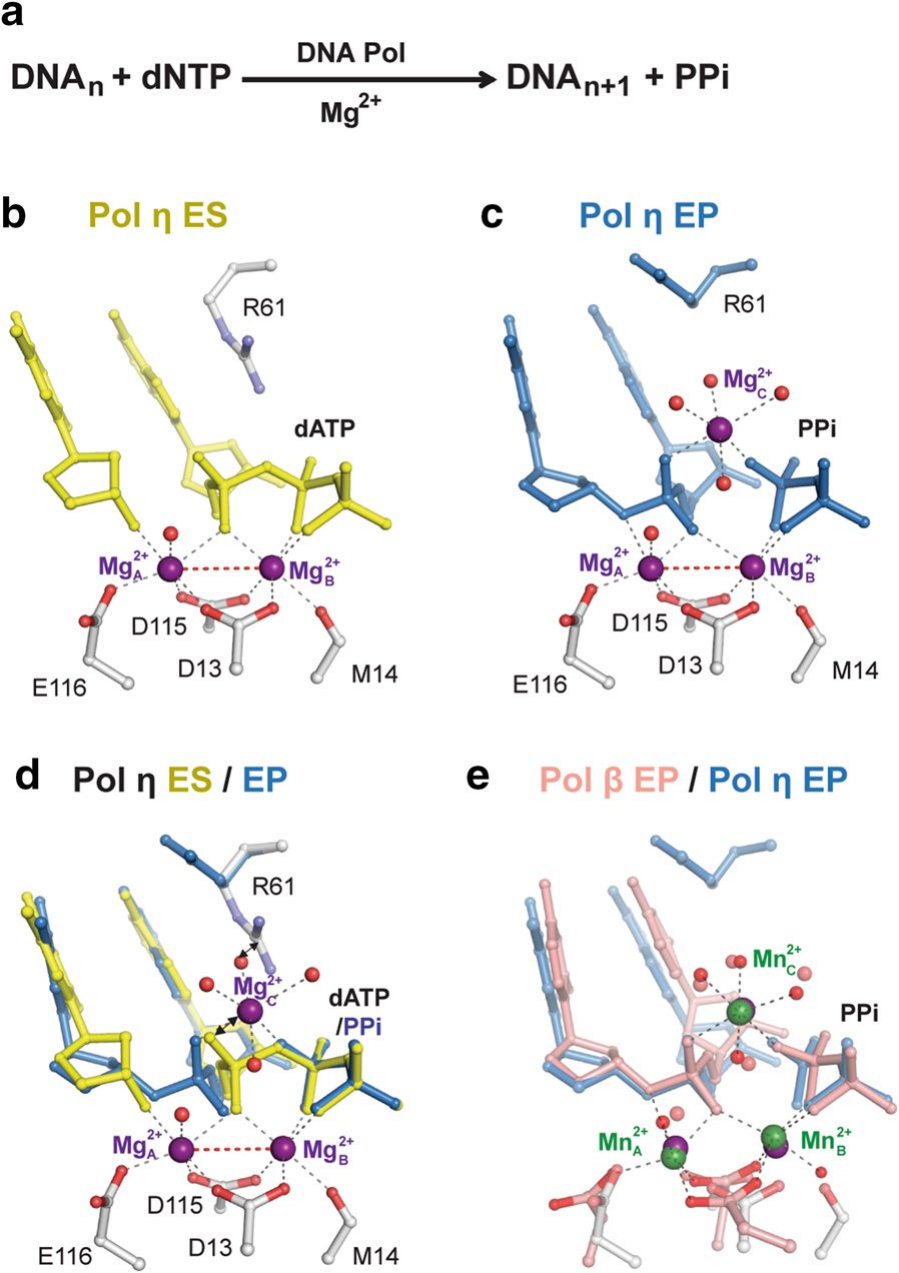
DNA synthesis reaction. **a** Chemistry of DNA synthesis reaction. **b** The structure of Pol η-DNA-dNTP (enzyme-substrate) complex (ES) replete with the canonical two metal ions. The B-site metal ion comes along with dNTP. Binding of the A site metal ion is greatly enhanced by a correct incoming dNTP, which forms a Watson–Crick base pair with the template base. With two canonical metal ions bound, the reactants 3′-OH and the α-phosphate of dNTP are perfectly aligned, but without the C-site metal ion no reaction can take place. **c** DNA Pol η in the enzyme-product complex (EP). The third metal ion occupies the C site and is coordinated by oxygen atoms from product DNA and pyrophosphate. The other four coordination ligands are water molecules, one of which may donate a proton to the pyrophosphate leaving group. **d** Co-existing ES and EP complexes of DNA Pol η. The third metal ion would clash with dNTP and is incompatible with the ES complex. The largest changes between ES and EP are the scissile phosphorus and the sugar pucker of the 3′-end nucleotide. **e** Superposition of DNA Pol η (*purple* Mg^2+^) and Pol β (*pink* and *green* Mn^2+^) in the enzyme-product complexes (EP). Despite different tertiary structures, the third metal ion (Mn^2+^) is also found in DNA Pol β in the same coordination geometry as in Pol η

Enzymes and associated catalytic reactions have been studied in the pre-steady and steady states to yield catalytic requirements, intermediate steps and rates at high temporal resolutions. DNA synthesis reactions are no exception. DNA polymerases catalyze phosphoryltransfer reactions that incorporate dNTPs (A, G, T and C) according to a template sequence one at a time into DNA primer (Fig. 1a; Additional file 1: Movie S1). The reaction is of S_N_2 type and is pH dependent. All DNA polymerases depend on the metal ions Mg^2+^ or Mn^2+^ for catalysis, but differ dramatically in their catalytic rates. Many DNA polymerases undergo dNTP-dependent large conformational changes, while some translesion polymerases do not [12, 26–30]. Extensive kinetic and FRET studies have shown that the large conformational changes in DNA polymerases are faster than the chemical reaction itself, and therefore the phosphoryltransfer reaction is the most critical step in all DNA polymerases [9, 31–33]. After decades of investigation, it remains uncertain how a 3′-OH nucleophile is activated and whether the rate-limiting step of DNA synthesis is chemical or conformational [12, 32, 34–37].

For high spatial resolution, a large number of DNA polymerases have been crystallized in complex with a DNA template and primer pair and incoming dNTP. Catalysis was invariably circumvented by removal of the nucleophilic 3′-OH (replaced by 3′-deoxyribose) at the primer end, substitution of Mg^2+^ or Mn^2+^ by Ca^2+^, which does not support the catalysis, or substitution of dNTPs by non-reactive ddNTP or dNMPNPP analogs [14–19, 38]. Despite the diverse tertiary structures, catalytic rates, and degrees of fidelity in DNA polymerases, the core of the active site contains two Mg^2+^ or Mn^2+^, which are coordinated by the conserved carboxylates, DNA and dNTP substrate in the same configuration [19, 38]. How catalysis takes place was not visualized except for by QM/MM (hybrid quantum mechanics/molecular mechanics) modeling [20–23], which never fails to show that products can form by rearranging protons, electrons and atoms.

Kinetic studies of DNA synthesis have also observed two different binding constants of Mg^2+^ [37]. But if more than one metal ion has a similar binding affinity, such metal-ion titration assays would lead to an under estimation. Because kinetic titration results agree with the number of metal ions found in the active site by X-ray crystallography [14–16, 26], two-metal-ion catalysis has become the widely accepted mechanism for DNA synthesis reactions. In the two-metal-ion catalysis model, one metal ion (B site) is associated with incoming dNTP and the second metal ion (A site) bridges the 3′-OH nucleophile and the incoming dNTP and is suggested to help deprotonating 3′-OH for the nucleophilic attack (Additional file 1: Movie S1). If incorrect or damaged, an incoming dNTP would be rejected before the A-site Mg^2+^ is recruited. Therefore, binding of two Mg^2+^ ions, which are notorious for the stringent coordination requirement, helps polymerases to achieve exceedingly high fidelity [39, 40]. As initially proposed by Steitz and Steitz [41], the DNA synthesis reaction is presumed to be promoted by the two Mg^2+^ ions, which align the substrates and facilitate the acid–base catalysis and the pentacovalent intermediate formation (Additional file 1: Movie S1).

### In crystallo catalysis of DNA synthesis

Because DNA synthesis is pH and metal ion dependent, by reducing pH to 6.0 and using the non-catalytic metal-ion cofactor Ca^2+^, crystals of native DNA Pol η in complex with DNA substrate and correct incoming dNTP can be grown over a couple of weeks without reaction taking place [24]. After fully grown, crystals were transferred to stabilization buffer to remove free reaction components and raise the pH to 7.0, which is permissible for the phosphoryltransfer reaction. To initiate DNA synthesis, crystals were exposed to Mg^2+^ or Mn^2+^, and after a certain reaction time they were cryo-cooled in liquid nitrogen to stop the reaction at the set time point. Ensuing analyses by X-ray diffraction have yielded unprecedented details following the reaction process of DNA synthesis [24]. Binding of the A and B site Mg^2+^ is necessary to fully align the 3′-OH of DNA primer and incoming dNTP. But only after a 40 s delay after two metal ion binding, does the chemical reaction take place [24].

Interestingly, in crystallo DNA synthesis has also been achieved with DNA polymerase β. Crystals of DNA polymerase β complexed with DNA substrate (binary complexes) have been grown; however, unlike our Pol η studies, the incoming dNTP and Ca^2+^ were soaked in later [42]. Similar to the studies of Pol η, catalysis was initiated by exposing Pol β crystals to Mg^2+^ or Mn^2+^ and terminated by cryo-cooling in liquid nitrogen. This approach has been applied successfully to study how Pol β incorporates an incorrect incoming nucleotide and oxidatively damaged 8-oxo-dG [42, 43]. Because the reaction rate of Pol β is much faster than Pol η, at the first time point of the data acquisition half of the substrate is already turned into product. Substrate alignment by two metal ions and the delayed chemical reaction were not observed with Pol β [42, 43].

### A third metal ion is essential for DNA synthesis

In both in crystallo catalysis by Pol η and β, a third divalent cation coordinated by reaction products is observed (Fig. 1b–d). In the Pol β case, the third metal ion appears at the first time point (20–30 s) when approximately half of the substrate was converted to products. With Pol η, whose reaction rate is much slower, the third Mg^2+^ was detected ~60 s after the initial appearance of products, when the products were accumulated to 40 % [24]. Because the third Mg^2+^ is coordinated by the DNA product and pyrophosphate leaving group and its binding is incompatible with the substrate state, it is dubbed “the product metal ion” [42, 43].

We were puzzled about the role of the third metal ion and wondered whether it is involved in catalysis or merely stabilizes the products and acts as a general acid to facilitate protonation of pyrophosphate [24]. Because of the low electron number of Mg^2+^, it is not possible to detect Mg^2+^ by X-ray diffraction if its occupancy is lower than 30 %. Therefore, even if the third Mg^2+^ is required for the catalysis and product formation, it is not detected until the amount of product reaches more than 30 %.

To detect the third metal ion at a low occupancy, we switched the reaction buffer from Mg^2+^ to electron-rich Mn^2+^. To our delight, Mn^2+^ not only allowed detection of the third metal ion at low occupancy, but the affinities for Mn^2+^ at the two canonical (A and B) and the third (C) sites are significantly different [25]. When in crystallo reaction took place at 10 mM Mn^2+^, the third Mn^2+^ appears at the same time as the reaction products and the two are perfectly correlated in appearance, time and occupancy. When the reaction was conducted in 1 mM Mn^2+^, however, the A and B sites were readily occupied, but the C-site was devoid of Mn^2+^. As a result, no products could be detected! Our in crystallo metal ion titration reveals the canonical A and B sites both have high-affinity for Mg^2+^ and Mn^2+^, and the C-site is of low affinity and thus determines the overall metal-ion concentration requirement for the DNA synthesis reaction [25, 37].

### Binding of the third metal ion is rate limiting

A protein sidechain, R61 of Pol η, forms bifurcated salt bridges with the α and β phosphates of the incoming dNTP in the reactant state and overlaps with the C-site metal ion in the polymerase-product complexes if R61 does not change its rotamer conformation [24]. One may imagine that removal of R61 would allow easier and faster binding of the third metal ion and lead to a faster reaction rate. When R61A mutation is introduced, however, the mutant Pol η catalyzes DNA synthesis at a slower rate than WT polymerase [19, 44, 45]. When the R61A mutant Pol η was examined in crystallo, we found that binding of the A and B site metal ions occurred within 40 s as for WT, but without R61 the incoming dNTP is misaligned relative to the 3′-OH by 0.3 Å. Consequently binding of the third metal ion was delayed by 120 s, thus leading to the slower catalytic rate than WT [25].

All DNA polymerases exhibited reduced catalytic rates when incoming dNTP is substituted with sulfur in the pro-Sp position (Sp-dNTPαS) [8, 34–36]. Before our discovery of the third metal ion, the Sp-sulfur was thought not to be involved in metal ion binding, and the degree of rate reduction has been interpreted as perturbing either chemistry or conformational change necessary for catalysis [46]. But the pro-Sp and the α,β bridging oxygen atoms of dNTP are the only two potential non-water ligands of the third metal ion (Fig. 1). We predicted that sulfur substitution of Sp-dNTPαS therefore would retard the third metal ion binding and reduce the reaction rate.

Experimentally we find that the concentrations of Mg^2+^ and Mn^2+^ needed for incorporating Sp-dNTP αS by Pol η in solution are increased by ten and threefold, respectively [25]. This is not surprising because the amount of Mg^2+^ or Mn^2+^ needed for the DNA synthesis reaction is determined by the C-site metal ion [25]. The most dramatic changes in the in crystallo reaction are slow product formation and absence of detectable third metal ion even in 20 mM Mg^2+^ or Mn^2+^. In addition, although not directly involved in the A and B site metal ion binding, the Sp-sulfur perturbs the occupancy and location of Mg^2+^ in the A site [25] probably due to an altered electrostatic environment. Because the reaction-rate reduction caused by Sp-dNTP αS is less than fourfold [35, 36], it was concluded that the rate-limiting step in DNA synthesis is conformational changes. But the high concentrations of Mg^2+^ (up to 12.5 mM) used in these assays, however, masked the severe defects of the C-site metal ion binding. If 1 mM of Mg^2+^ had been used, the rate reduction would have been much greater, and thus leading to the opposite conclusion that chemistry is the rate-limiting step!

Our study reveals that binding of the C-site metal ion is essential and that its occurrence determines when the chemical reaction takes place. Binding of the two canonical metal ions at the A and B sites is a prerequisite for capturing the third metal ion but it is not rate limiting. Because delays of the C-site metal ion binding reduce the reaction rate as observed with R61A Pol η and Sp-dNTPαS substitution, we conclude that binding of the third divalent cation is the rate-limiting factor in the DNA synthesis reaction.

### Solving the conundrum of when and how the third metal ion binds

The requirement of the third metal ion for the DNA synthesis reaction and its absence in the enzyme-substrate state present a conundrum—when and how the third metal ion binds. With nothing else to turn to, we hypothesized that thermal energy and thermal motion of the well-aligned reactants might provide a transient entrance for the third metal ion. To test the hypothesis, we divided the DNA synthesis reaction into two stages, A- and B-site metal ion binding along with alignment of DNA and dNTP substrate as the first, and capture of the third metal ion and product formation as the second. The first stage was carried out at 1 mM Mn^2+^. The second stage was conducted in the presence of 5 mM Mn^2+^ at various temperatures. Variation from 4 to 37 °C did not alter the diffusion rate of Mn^2+^ as evident in its unchanged binding at the canonical A site, but the increased thermal motions significantly enhanced the third metal ion binding and product formation [25].

**Fig. 2:**
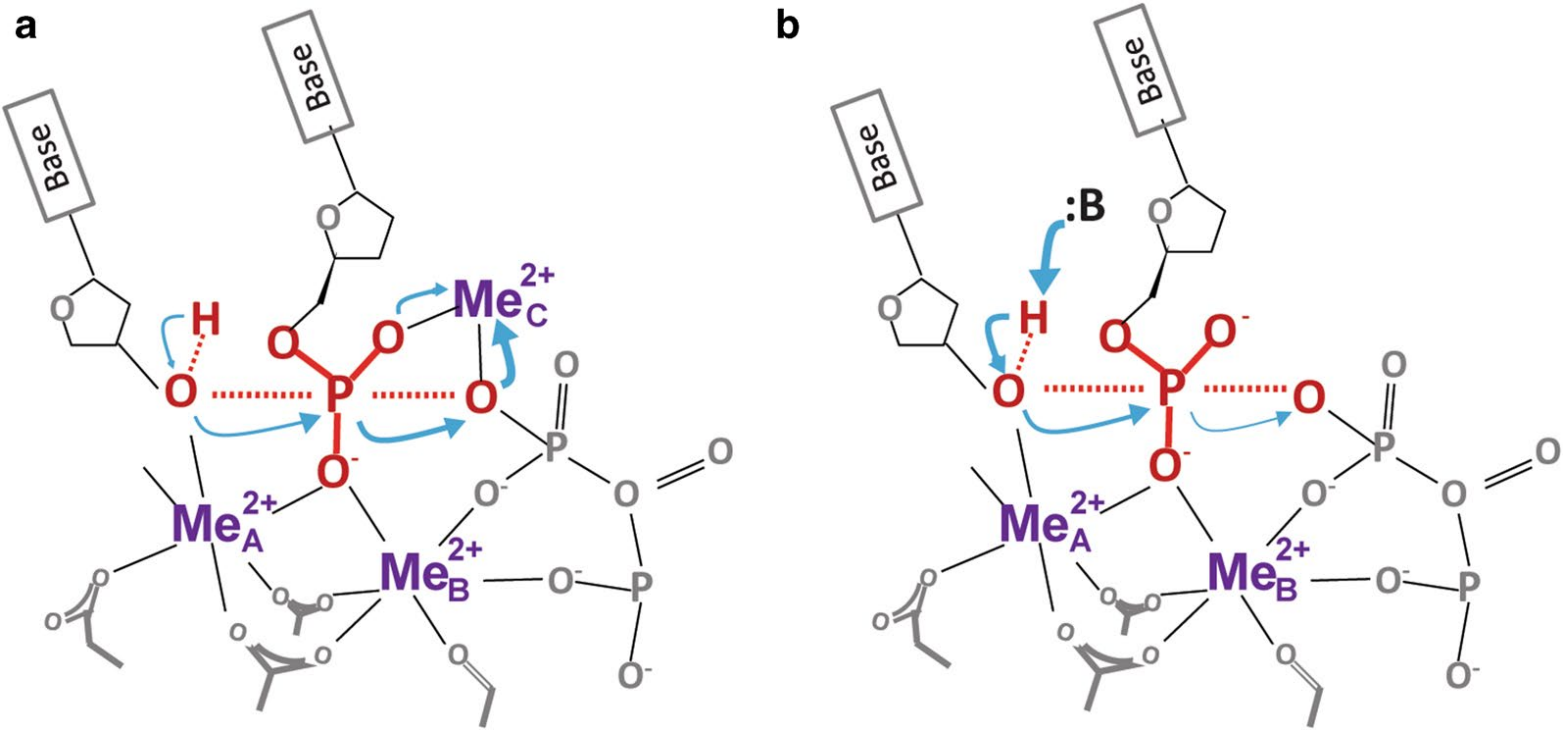
Comparison of the initiation of phosphoryltransfer in two- versus three-metal-ion catalysis. **a** In three-metal-ion catalysis, the C-site metal ion initiates the reaction by breaking the existing phosphodiester bond in dNTP and thus drives the phosphoryltransfer reaction. A well-aligned native 3′-OH is required for capture of the C-site metal ion and its deprotonation is a result of the reaction. **b** In two-metal-ion catalysis, the reaction starts by de-protonation of the 3′-OH (nucleophile), which activates nucleophilic attack and leads to breakage of the existing phosphodiester bond in dNTP

The coordination of the third metal ion by two oxygen atoms of an incoming dNTP is non-ideal in distance and geometry compared to the preferred octahedral geometry of six inner-shell ligands of Mg^2+^ ion. But its coordination in the product state, when the phosphodiester bond between the α and β phosphates is broken, is nearly perfect octahedral. We suspect that the free- energy gain from binding of the third Mg^2+^ ion (freeing two inner-shell water ligands and association with dNTP) clears the barrier to the transition state. The stringent preference for octahedral coordination by Mg^2+^ ion may also drive the phosphotransfer reaction from breaking the existing bond in dNTP to forming a new bond between the 3′-OH and the α phosphate, which is in the reverse direction of the standard textbook version that is starting from the nucleophilic attack to the bond breakage (Fig. 2).

## Conclusions

Comparison of the existing DNA polymerases, reverse transcriptases and RNA polymerases demonstrates that the active site compositions are highly conserved. Because both DNA pol η and pol β, which differ in tertiary structures, require the conserved third metal ion in catalysis, we propose that the third metal ion is a general feature and all polymerization reactions of nucleic acids occur by three-metal-ion catalysis (Additional file 2: Movie S2). The different environment surrounding the third metal ion, which is in a stark contrast to the conserved environment surrounding the canonical A and B site metal ions, gives hope that the third metal ion binding can be targeted for species and enzyme specific inhibition of DNA polymerases in treating infectious diseases and cancers.

**Fig. 3:**
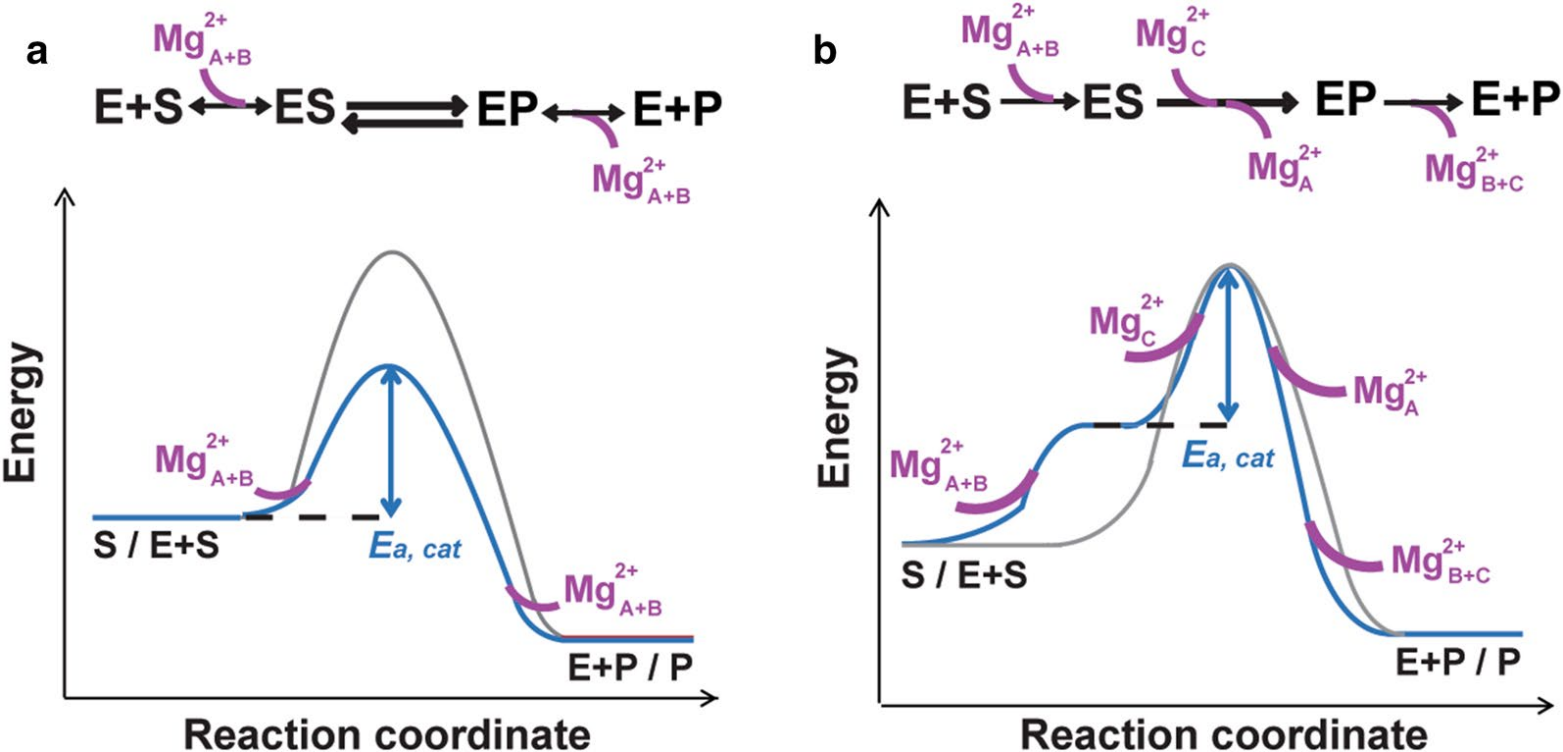
Modification of the transition-state theory. **a** The transition state theory suggests (*1*) a quasi-equilibrium between the substrate and transition state, which have the same chemical components, and (*2*) enzyme accelerating reaction rate by stabilizing transition state and thus reducing the energy barrier. The uncatalyzed and enzyme-catalyzed reaction processes are shown in *grey* and *blue*, respectively. We find that the canonical two Mg^2+^ ions are insufficient to support DNA synthesis. **b** Finding a third Mg^2+^ ion necessary for catalysis leads us to propose a new paradigm. The enzyme helps reaction substrates to capture the additional divalent cation and obtain necessary energy for transition from the substrate to product state

Extended from kinetic and structural studies, which lay the ground work for understanding DNA synthesis reactions, our in crystallo reaction and time resolved X-ray diffraction analysis has led to the discovery of the third metal ion and its capture being the rate-limiting step of DNA synthesis reaction. This finding reveals the different chemical compositions between the transition state and the reactant state and therefore modifies the transition-state theory of enzyme catalysis. The third metal ion, which is free of enzyme coordination, is probably needed for the phosphoryltransfer reaction with or without enzyme catalysis. The catalytic role of DNA polymerases may not be to reduce the energy barrier (Fig. 3a), which would increase both forward and reverse reaction rates, but rather is to align the substrates perfectly thus increasing the probability for an incoming dNTP to capture the third and the catalytic divalent cation thus overcoming the barrier to product formation (Fig. 3b).

## Additional files

10.1186/s13578-016-0118-2 Animation of the classic two-metal-ion catalysis of DNA synthesis. Two canonical metal ions are bound when an incoming dNTP forms Watson–Crick base pair. Binding of the two metal ions aligns the reactants, the 3′-OH and the α-phosphate of dNTP. The reaction proceeds by deprotonating the 3′-OH (nucleophile) by the general base and nucleophilic attack leads to formation of the pentacovalent bipyramid scissile phosphate intermediate. Bond breakage between the α- and β-phosphate is followed by protonation of the pyrophosphate leaving group by the general acid. How the substrates are activated to form the transition state is unknown, and which particular groups serve as the general base and acid for proton transfer is also unknown.
10.1186/s13578-016-0118-2 Animation of the new paradigm: the three-metal-ion catalysis of DNA synthesis. Thermal motion of the well-aligned reactants allows the entrance of the third metal ion, and the free energies generated from its binding to the reactants overcome the barrier to transition state and product formation. The proton-leaving path is varied (unpublished results of M. R. Gregory and W. Yang) and there is no specific general base. One of the four water ligands of the third metal ion likely serves as a proton donor (general acid) to the pyrophosphate leaving group.

## Abbreviation

TST: transition state theory
