# Erratum to: A new paradigm of DNA synthesis: three-metal-ion catalysis

**DOI:** 10.1186/s13578-017-0159-1

**Published:** 2017-06-20

**Authors:** Wei Yang, Peter J. Weng, Yang Gao

**Affiliations:** Laboratory of Molecular Biology, NIDDK, National Institutes of Health, 9000 Rockville Pike, Bethesda, MD 20892 USA

## Erratum to: Cell Biosci (2016) 6:51 DOI 10.1186/s13578-016-0118-2

Upon publication of the original article [1], it was noticed that the Fig. 2 is incorrect. This has now been acknowledged and corrected in this erratum. The correct Fig. 2 has been shown below.

**Fig. 2:**
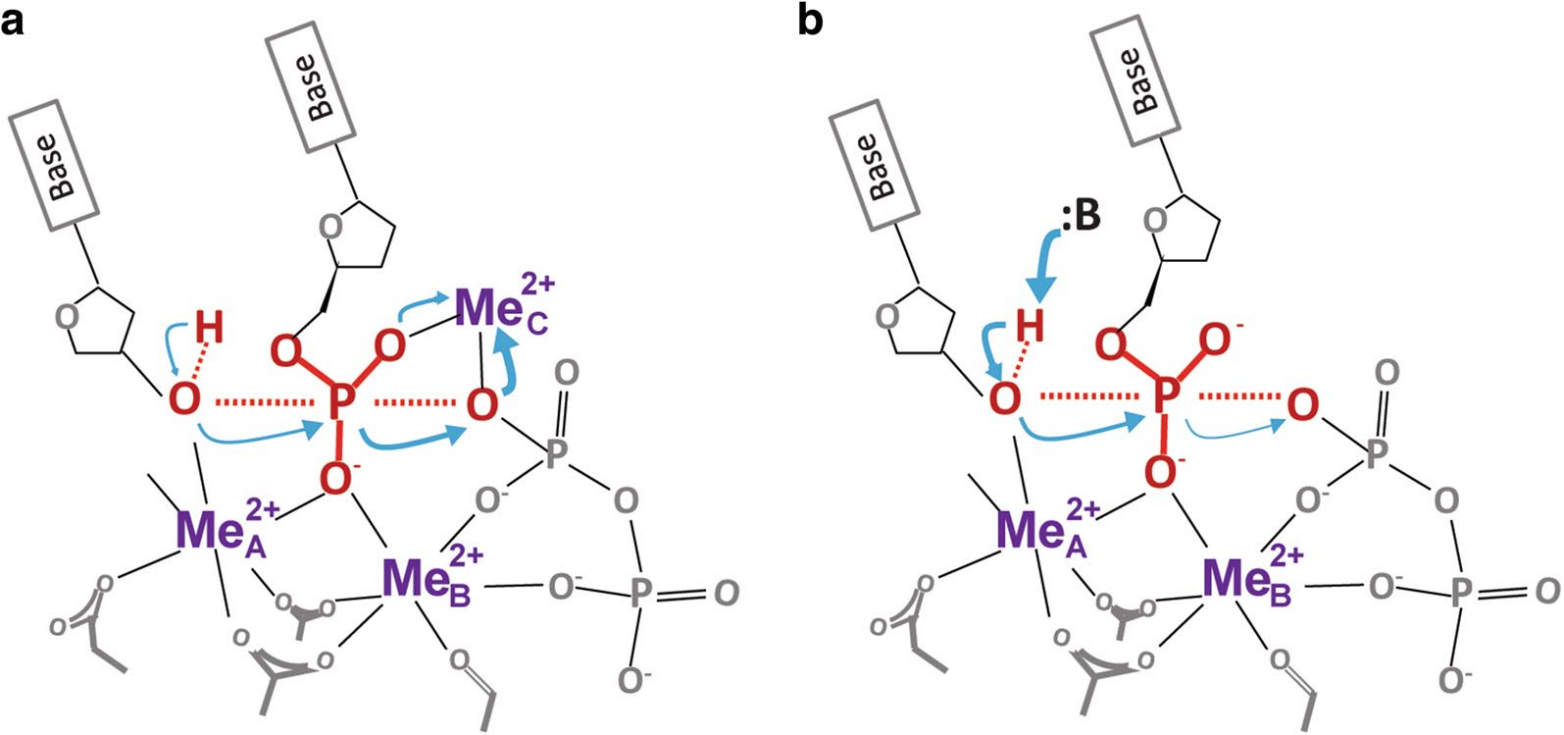
Comparison of the initiation of phosphoryltransfer in two- versus three-metal-ion catalysis. **a** In three-metal-ion catalysis, the C-site metal ion initiates the reaction by breaking the existing phosphodiester bond in dNTP and thus drives the phosphoryltransfer reaction. A well-aligned native 3′-OH is required for capture of the C-site metal ion and its deprotonation is a result of the reaction. **b** In two-metal-ion catalysis, the reaction starts by de-protonation of the 3′-OH (nucleophile), which activates nucleophilic attack and leads to breakage of the existing phosphodiester bond in dNTP
